# Investigation of Misleading Techniques Used in Online Fluoride Misinformation

**DOI:** 10.1111/idh.70053

**Published:** 2026-04-08

**Authors:** Matheus Lotto, Caio Sampaio, Olívia Santana Jorge, Juliano Pelim Pessan, Thiago Cruvinel

**Affiliations:** ^1^ Department of Pediatric Dentistry, Orthodontics, and Public Health, Bauru School of Dentistry University of São Paulo Bauru Brazil; ^2^ Department of Preventive and Restorative Dentistry, School of Dentistry São Paulo State University (UNESP) Araçatuba Brazil

**Keywords:** eHealth, fluoride, infodemiology, misinformation, social media

## Abstract

**Objectives:**

Fluoride misinformation can fuel anti‐fluoridation movements, influencing political decisions and dental treatment choices. This study aimed to elucidate the misleading techniques used to produce online fluoride misinformation.

**Methodology:**

Two independent investigators searched scientific databases for references that supported or refuted 33 fluoride misinformation messages, derived from a prior study that manually identified dental falsehoods on websites through thematic content analysis. The levels of evidence were assessed according to the Oxford Centre for Evidence‐Based Medicine (levels 1–5) and the GRADE guidelines (high to very low, indicating the degree of confidence that the direction of the net effect is correct). Misleading techniques were classified as false attribution, when no reference supported a message, or quote mining, when at least one supporting reference was identified but used out of context.

**Results:**

Nineteen references were identified to refute 27 fluoride misinformation, while seven references were identified to support six misinformation messages. False attribution was the predominant technique (81.82%), compared to quote mining (18.18%). The levels of evidence and strength of recommendations for refuting references ranged from 1A–1B (high, 31.6%) to 2C–5 (very low, 57.9%). Supporting references ranged from 2B (low, 14.3%) to 2C–5 (very low, 85.7%). The claim ‘fluoride accumulates in the body’ was the only one supported by low‐level evidence.

**Conclusions:**

Online fluoride misinformation is most often grounded in false attribution. Understanding the strategies that authors use to construct these false messages can support health educators in creating more targeted and effective prebunking and debunking interventions.

## 1 Introduction

It is noteworthy the key role of contextual factors in shaping digital content [1], especially in today's post‐truth era, where personal beliefs are usually given more importance than scientific evidence [2, 3]. In this environment, people tend to engage with online content that confirms their existing health beliefs, often without questioning its accuracy [4, 5]. As a result, they become more vulnerable to manipulated information spread by agents with hidden interests [6, 7]. One clear example is the increasing hesitation around fluoride use, which is largely based on hypothetical side effects that lack strong scientific support [8, 9, 10].

This study was driven by the increasing dissemination of online fluoride misinformation. Within this context, the manipulation of digital content to mislead audiences aligns with the principles outlined in Information Manipulation Theory [11, 12]. It posits that deception often involves subtle distortions of truthful information rather than the outright fabrication of falsehoods. It identifies four primary forms of deceptive communication: withholding relevant information, emphasizing trivial or irrelevant details, disseminating false claims, and using vague or ambiguous language [12]. The effectiveness of these tactics relies heavily on the deception going undetected by the audience [11, 12]. Consequently, misinformation spreaders frequently employ these strategies to enhance the perceived credibility of their messages, thereby fueling the circulation of misleading content [13].

Although misleading arguments can take various forms, a widely accepted definition characterizes them as deductively invalid, inductively weak, based on unjustified premises, or dismissive of relevant evidence available to the speaker [14]. In the realm of online health misinformation, false attribution is a frequent strategy, occurring when a scientific reference is misused—such as citing a source that is irrelevant, unqualified, unidentified, biased or even fabricated—to lend false credibility to a claim [15]. Another common tactic, known as quote mining, involves extracting a statement from its original context in order to misrepresent its intended meaning and falsely reinforce a message [16]. For instance, claims about fluoride toxicity may hold some truth in cases of excessive ingestion; however, such assertions must be contextualized, particularly in light of fluoride's well‐established benefits in preventing dental caries when used appropriately in public health settings. Understanding these deceptive techniques is therefore critical for developing effective countermeasures against the spread of fluoride‐related misinformation.

Therefore, this study aimed to identify the misleading techniques used in online fluoride misinformation, with a focus on its referential background and level of evidence. We hypothesized that the content of fluoride misinformation messages is determined by the quality or presence of scientific evidence (*H*_1_). This study draws on Wood's framework, which highlights the role of individuals' cognitive goals, motivations, and biases—such as fear, distrust, or the desire to align with a particular worldview—as relevant factors in the production of misleading narratives. In this view, reasoning is shaped by personal characteristics strongly influenced by the reasoner's cognitive agenda [17].

## 2 Study Population and Methodology

The authors followed the recommendations of the Standards for Reporting Qualitative Research (SRQR) [18].

### 2.1 Study Design

This study was designed to systematically evaluate and classify the level of evidence associated with fluoride misinformation messages [19] using a rigorous, multi‐step process. Two experienced researchers (J.P.P. and C.S.) were responsible for identifying references that supported or refuted fluoride misinformation messages. They also evaluated the levels of evidence for these references using the Oxford Centre for Evidence‐Based Medicine (OCEBM) criteria [20] (Phase I). Subsequently, two additional investigators (T.C. and M.L.) converted the OCEBM levels into certainty of evidence levels according to the GRADE (Grading of Recommendations, Assessment, Development, and Evaluations) framework [21]. In addition, they determined the misleading techniques employed to produce fluoride misinformation messages (Phase II) (Figure 1).

**FIGURE 1:**
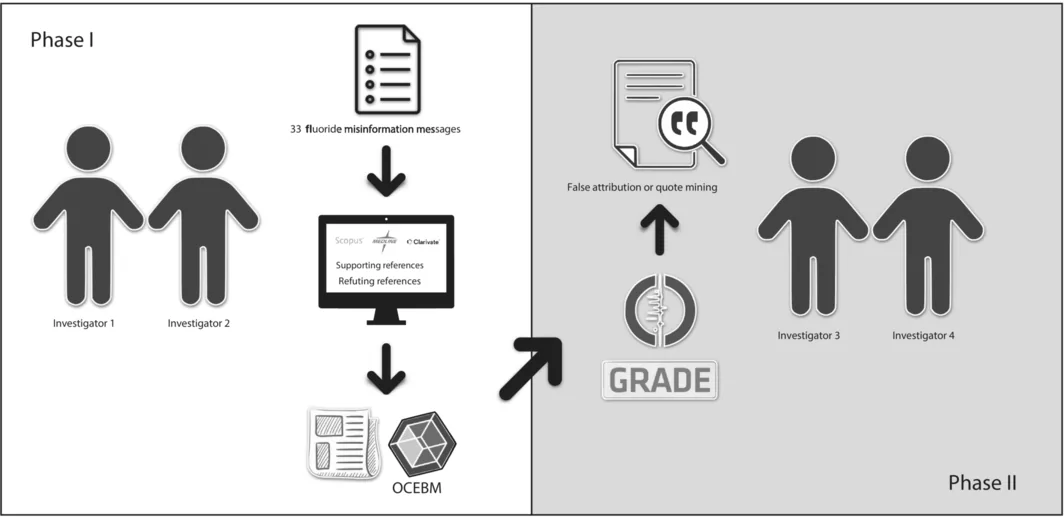
Visual summary of the study design.

### 2.2 Ethics

This study did not require approval from the Ethics Committee on Human Research of the Bauru School of Dentistry, University of Sao Paulo. This exemption was due to the use of publicly available data, which does not involve direct interaction with individuals or pose risks to participants [22].

### 2.3 Researcher Characteristics and Reflexivity

J.P.P. and C.S. have conducted multiple research projects focused on developing technologies to optimize and expand the benefits of fluoride for public health. All researchers involved in the present study are also strongly aligned with the current body of evidence supporting the use of fluoride in collective and individual strategies for the prevention of dental caries.

### 2.4 Context

The fluoride misinformation messages that originated this study were identified in a primary research project [19], in which two independent investigators (M.L. and O.S.J.) analysed 410 websites ranked as most relevant by Google for specific oral health content. They manually identified distinct pieces of misinformation using thematic content analysis.

This research must be contextualized within a specific space and time. It was conducted by researchers affiliated with Brazilian universities. Brazil has mandated water fluoridation as a preventive public health measure since the 1950s, with strong consensus among dental researchers supporting its continued use. It is important to note that dental caries remains highly prevalent and incident in Brazil, particularly among underserved populations. The study was performed during a period marked by intensified social debates opposing collective preventive measures, such as water fluoridation, often fueled by denialist arguments about the risks and benefits of fluoride for the prevention of dental caries.

### 2.5 Sampling Strategy

Independent comprehensive searches were performed across Medline, Scopus and Web of Science databases to identify and collect references that either support or refute 33 fluoride misinformation messages. The references were selected regarding the highest level of evidence, to ensure that the analysis was grounded in the most credible and robust scientific data available.

### 2.6 Data Collection Methods, Instruments and Technologies

The investigators recorded data from the collected references using independent Excel spreadsheets (Microsoft, Redmond, USA). These sheets included the following information: authors' names, year of publication, type of reference (refuting or supporting), fluoride misinformation messages associated with the reference, and level of evidence.

An iterative process was used for data collection. When each investigator considered their search complete independently, they discussed their findings to determine whether the task was finalized or required continuation. This process was repeated until both investigators agreed that the searches were satisfactory.

### 2.7 Units of Study

Each paper identified as a supporting or refuting reference to a fluoride misinformation message was considered a unit of analysis.

### 2.8 Data Processing

Data integrity was verified through double‐checks conducted by independent pairs of investigators—during data collection (J.P.P. and C.S.) and after its completion (T.C. and M.L.).

### 2.9 Data Analysis

Two investigators (J.P.P. and C.S.) employed the OCEBM guidelines [20] to scrutinize the identified references and ascertain their level of evidence, choosing the article with the highest evidence level to demonstrate support or refutation of a specific fluoride misinformation message. The OCEBM organizes evidence into five levels (from 1 to 5), with level 1 representing the highest‐quality studies and level 5 representing the lowest‐quality studies. These levels are based on study design, methodological rigour, and relevance to the clinical question. Specifically, level 1 includes systematic reviews of randomized controlled trials (RCTs) or high‐quality individual RCTs with narrow confidence intervals. Level 2 comprises lower‐quality RCTs, prospective cohort studies, or systematic reviews of such studies. Level 3 includes case–control studies or systematic reviews of case–control studies. Level 4 refers to case series and poor‐quality cohort or case–control studies, and level 5 is based on expert opinion without explicit critical appraisal or on basic science research (‘first principles’).

The OCEBM framework is adaptable to different types of clinical questions—therapy, diagnosis, prognosis and aetiology/harm—with specific criteria tailored to each domain. Importantly, it emphasizes critical appraisal beyond simple categorization: investigators are encouraged to assess study validity, consistency of results, directness of evidence, precision and potential biases when assigning a level [20].

Subsequently, two additional investigators (T.C. and M.L.) converted the levels of evidence into certainty of evidence ratings according to the GRADE scale, as described by Klugar et al. [21]. The GRADE framework offers a systematic method for rating the certainty of evidence across studies, complementing and extending the OCEBM hierarchical classification. In this process, higher OCEBM levels (e.g., level 1) are typically associated with high certainty of evidence in GRADE, while lower levels (e.g., levels 4 and 5) correspond to low or very low certainty. However, this mapping is not purely mechanical; it involves careful consideration of additional GRADE domains such as risk of bias, inconsistency, indirectness, imprecision, and publication bias, which may result in downgrading or upgrading the certainty ratings [21].

More specifically, level 1 evidence (e.g., systematic reviews of RCTs) generally starts at high certainty; levels 2 and 3 (e.g., cohort or case–control studies) typically begin at moderate or low certainty, depending on methodological robustness; and levels 4 and 5 (e.g., case series, expert opinion) are usually assigned low or very low certainty from the outset. While OCEBM provides a preliminary categorization based on study design and quality, the GRADE approach demands a comprehensive, transparent judgement that evaluates the relevance and robustness of the evidence. Thus, the conversion ensures that the final certainty of evidence reflects not only the type of study design but also its broader trustworthiness and applicability [20, 21].

Finally, this process enabled the same investigators from the primary study (T.C. and M.L.) to identify the misleading techniques used by authors in constructing false fluoride messages, as they were familiar with the original contexts in which the sentences appeared. For example, the statement ‘fluoride accumulates in the body’ is factually correct; however, it may be presented in a misleading context, such as ‘fluoride is dangerous and must be avoided since it accumulates in the body’. The strategies identified included false attribution, defined as the absence of relevant sources supporting the misleading argument, and quote mining, in which a legitimate source is misused to support an argument taken out of its original evidentiary context [12, 15, 16]. In the example above, the misleading technique would be classified as quote mining, as there is no scientific evidence indicating that fluoride accumulation, when used according to clinical recommendations, poses a health risk.

In all instances of divergent classifications regarding OCEBM levels, GRADE assessments, or identification of misleading techniques, the investigators systematically discussed discrepancies to reach a consensus.

### 2.10 Techniques to Enhance Trustworthiness

A decontextualized analysis of fluoride misinformation messages, double data verification, independent and repeated classification of evidence levels at different time points, consensus building among investigators, and the use of two distinct frameworks for evaluating evidence were employed to strengthen the trustworthiness of the findings.

## 3 Results

Table 1 summarizes the levels of evidence of references that support or refute fluoride misinformation messages.

**TABLE 1:** Comprehensive evaluation of supporting and refuting references on fluoride messages—unveiling the level of evidence and identifying misleading techniques employed by authors to disseminate misinformation.

References	Type of reference	Misinformation	Evidence level		Misleading techniques
			Oxford	Grade	
Belotti and Frazão [9]	Refuting	The studies that launched fluoridation were methodologically flawed	3A	Very low	False attribution
CDC [23]	Supporting	The chemicals used to fluoridate water are not pharmaceutical grade	5	Very low	Quote mining
Fishta et al. [24]	Supporting	Fluoride may cause reproductive problems	3A	Very low	Quote mining
		There is no margin of safety for fluoride use for several health effects			
Health National Foundation [25]	Supporting	The chemicals used to fluoridate water are not pharmaceutical grade	5	Very low	Quote mining
Lidbeck et al. [26]	Supporting	Fluoride is used as a poison	5	Very low	Quote mining
		There is no margin of safety for fluoride use for several health effects			
Luke [27]	Supporting	Fluoride affects the pineal gland (calcification of the pineal gland)	5	Very low	Quote mining
Maas et al. [28]	Supporting	Fluoride may leach lead from pipes, brass fittings, and soldered joints	5	Very low	Quote mining
Eble et al. [29]	Supporting	Fluoride accumulates in the body	2B	Low	Quote mining
Clarkson et al. [30]	Refuting	Proponents usually refuse to defend fluoridation in open debate	5	Very low	False attribution
Iheozor‐Ejiofor et al. [31]	Refuting	Fluoride is an artificial chemical and therefore bad for consumption	3A	Very low	False attribution
		Minorities are not being warned about their vulnerabilities to fluoride			
		Proponents use very dubious tactics to promote fluoridation			
		Water fluoridation is a communist plot to control minds			
		Water fluoridation was designed to boost the sugar lobby			
		There is no margin of safety for fluoride use for several health effects			
Kaipio et al. [32]	Refuting	Fluoride may cause heart disease	2C	Very low	False attribution
		There is no margin of safety for fluoride use for several health effects			
Kim et al. [33]	Refuting	Fluoride may cause osteosarcoma	3B	Very low	False attribution
		There is no margin of safety for fluoride use for several health effects			
National Toxicology Program [34]	Refuting	Fluoride may cause autism	3A	Very low	False attribution
		Fluoride may cause poor memory			
		Fluoride may cause ADHD			
		Fluoride may lower IQs in children			
		Fluoride may cause neurotoxicity			
		Fluoride may damage the brain			
		Fluoride may cause Alzheimer's disease			
		There is no margin of safety for fluoride use for several health effects			
Oweis et al. [35]	Refuting	Dental fluorosis may be an indicator of broader systemic damage	2C	Very low	False attribution
		There is no margin of safety for fluoride use for several health effects			
Shaik et al. [36]	Refuting	Fluoride affects thyroid function	3B	Very low	False attribution
		There is no margin of safety for fluoride use for several health effects			
Sowanou et al. [37]	Refuting	Fluoride causes arthritis symptoms	3B	Very low	False attribution
		There is no margin of safety for fluoride use for several health effects			
Yang et al. [38]	Refuting	Fluoride may cause cancer	2C	Very low	False attribution
		There is no margin of safety for fluoride use for several health effects			
Wang et al. [39]	Refuting	Fluoride kills gut bacteria	3B	Very low	False attribution
Saylor et al. [40]	Refuting	Some individuals are highly sensitive to low levels of fluoride	2B	Low	False attribution
Yin et al. [41]	Refuting	Fluoride may increase hip fractures in the elderly	2A	Low	False attribution
		There is no margin of safety for fluoride use for several health effects			
Lin et al. [42]	Refuting	Fluoride is an artificial chemical and therefore bad for consumption	1A	High	False attribution
		Minorities are not being warned about their vulnerabilities to fluoride			
		Fluoride damages bone			
		Proponents use very dubious tactics to promote fluoridation			
		There is no margin of safety for fluoride use for several health effects			
Macek et al. [43]	Refuting	The silicon fluorides have not been tested comprehensively	1B	High	False attribution
		The silicon fluorides may increase lead uptake into children's blood			
Marinho et al. [44]	Refuting	Fluoride is an artificial chemical and therefore bad for consumption	1A	High	False attribution
		Proponents use very dubious tactics to promote fluoridation			
		There is no margin of safety for fluoride use for several health effects			
Marinho et al. [45]	Refuting	Fluoride is an artificial chemical and therefore bad for consumption	1A	High	False attribution
		Proponents use very dubious tactics to promote fluoridation			
		There is no margin of safety for fluoride use for several health effects			
Marinho et al. [46]	Refuting	Fluoride is an artificial chemical and therefore bad for consumption	1A	High	False attribution
		Proponents use very dubious tactics to promote fluoridation			
		There is no margin of safety for fluoride use for several health effects			
Walsh et al. [47]	Refuting	Fluoride is an artificial chemical and therefore bad for consumption	1A	High	False attribution
		Proponents use very dubious tactics to promote fluoridation			
		There is no margin of safety for fluoride use for several health effects			

The investigators identified a total of 26 references. Six fluoride misinformation messages (18.18%) were supported by seven references, of which six had a very low level of evidence [23, 24, 25, 26, 27, 28] and one had a low level of evidence [29]. The content of these messages was primarily based on mechanism‐based reasoning studies [27, 28], a type of study in which conclusions are drawn from understanding biological, physiological or social mechanisms (e.g., ex vivo and in vitro studies)—rather than from directly observing outcomes in humans—as well as expert opinions [23, 25], a case series report [26], a cross‐sectional study [29], and a systematic review of case–control studies [24].

On the other hand, 27 fluoride misinformation messages (81.82%) were refuted by 19 references, with the level of evidence varying across very low [9, 30, 31, 32, 33, 34, 35, 36, 37, 38, 39], low [40, 41], and high [42, 43, 44, 45, 46, 47]. These references encompassed mechanism‐based reasoning studies [34, 36], expert opinions [30], case–control studies [33, 37], ecological studies [32, 36, 38, 43], a retrospective cohort study [40], a prospective cohort study [35], systematic reviews of observational studies [9, 31, 41], and systematic reviews of randomized clinical trials [42, 44, 45, 46, 47].

Consequently, the technique of false attribution was identified in 27 fluoride misinformation messages (81.82%), while the technique of quote mining was identified in six messages (18.18%). These findings underscore that the majority of fluoride misinformation messages analysed in this study were fabricated without any connection to factual evidence, as no sources were found to support the misleading arguments.

## 4 Discussion

To our knowledge, this study is the first to explore the misleading techniques behind online fluoride misinformation, examining its sources and levels of evidence. Most fluoride misinformation messages were found to rely on false attribution. While some messages were linked to references based on studies with high levels of evidence, there was a recurring dependence on sources of very low quality. Messages constructed through quote mining were exclusively supported by references classified as low or very low level of evidence. Therefore, hypothesis *H*_1_ was partially confirmed.

Those spreading misinformation about fluoride often cite scientific references to discourage fluoride use, yet they frequently overlook the limitations of the studies they reference. For example, the claim that “fluoride affects the pineal gland” is based on a post‐mortem study showing fluoride accumulation in the pineal glands of elderly individuals (mean age 82 years, range 70–100) [27]. However, this study does not establish a causal link between fluoride exposure and health outcomes, mainly due to its observational design. Similarly, a portion of misinformation messages were based on outcomes from in vitro studies [28] to argue against fluoride use. Other messages rely on expert opinions [23, 25], criticizing the lack of pharmaceutical‐grade chemicals in fluoridation programs Similar arguments could equally apply to other water treatment substances like chlorine and ozone.

Additionally, a portion of misinformation messages distorted accidental high‐dose fluoride ingestion cases [26] or cross‐sectional findings linking fluoride concentration in bones with age [29], ignoring established concepts of safe exposure levels and the physiological accumulation of fluoride in mineralized tissues. There are also examples of extrapolations from systematic reviews of case–control studies, suggesting associations between fluoride consumption and female reproductive health issues, despite important limitations in study design [24].

When analysing references that refute misinformation, most were also based on low‐ to very low‐level evidence from retrospective cohort and ecological studies. Conducting large clinical trials to confirm weak hypotheses identified in observational studies and expert opinions (e.g., claims that ‘fluoride may cause cancer’ or ‘fluoride may cause heart disease’) remains impractical [9, 30, 31, 32, 33, 34, 35, 36, 37, 38, 39]. However, references based on stronger evidence clearly disprove misleading claims. For instance, the assertion that ‘fluoride is an artificial chemical and therefore harmful’ is contradicted by multiple randomized clinical trials that consistently demonstrated the importance of fluoride in preventing dental caries across different populations and age groups [42, 43, 44, 45, 46, 47].

These findings underscore an important challenge for the development of technologies aimed at countering fluoride misinformation. Effectively addressing this issue requires a nuanced understanding of the context in which messages are produced and disseminated. Although most misinformation messages were overtly fabricated, approximately 18% of the messages demanded a more detailed examination to identify subtle manipulative strategies of knowledge. In this regard, artificial intelligence tools—particularly deep learning models—hold considerable promise for detecting misinformation across diverse contexts. By incorporating the recognition of misleading techniques into AI frameworks, it becomes possible to leverage their capabilities in contextual interpretation, semantic accuracy, generalization, and adaptability, enhancing their effectiveness in identifying and mitigating the spread of fluoride‐related misinformation across digital platforms [48, 49]. Accordingly, the present outcomes contribute valuable guidance for research initiatives seeking to automate the detection of fluoride misinformation in large‐scale datasets, minimizing reliance on manual content analysis and improving the efficiency of efforts to address information pollution.

Previous studies have mainly focused on describing online fluoride misinformation [7, 50, 51, 52, 53], without systematically analysing the underlying narratives or assessing the quality of supporting evidence. These studies have emphasized users' concerns about fluoride, often fueled by speculative hypotheses about potential side effects [7, 50, 51, 52, 53]. This fear leads to a reduced perception of the risk of dental caries and an increased interest in unreliable fluoride content, especially in countries with lower rates of untreated dental caries [53, 54].

Moreover, the current ‘post‐truth’ environment—where personal beliefs are often prioritized over scientific evidence [2, 3]—has intensified the dissemination of fluoride misinformation. Individuals increasingly cite references to reinforce their preexisting health‐related views, regardless of the scientific rigour of those sources. Political motivations also contribute to the spread of unreliable information [55, 56]. Anti‐fluoridation narratives frequently intersect with political agendas, given that public water fluoridation policies are closely linked to government‐led public health initiatives [17]. Consequently, online debates surrounding fluoridation have influenced real‐world behaviours, such as fluoride hesitancy and organized community efforts to discontinue water fluoridation programs [8, 57, 58]. Issues and queries that associate fluoride with political themes, such as conspiracies involving Adolf Hitler, indicate user engagement with content aligned to their ideological beliefs and values, often at the expense of critically assessing scientific evidence [53]. In this context, politically motivated misinformation may be strategically employed by lobbying groups and political movements to erode trust in scientific expertise and further specific agendas. These dynamics are amplified by partisan motivated reasoning, in which individuals exhibit greater trust in information presented by ideologically aligned sources [59]. As a result, trust in legitimate scientific information is compromised, fostering polarized attitudes and reducing adherence to evidence‐based preventive health measures.

These findings should be interpreted with caution. First, the primary study may not have captured all existing online fluoride misinformation messages, as the results were shaped by the choices made during data collection. However, a large amount of online content was analysed, and thematic saturation was reached to address this limitation. Second, the analysis was limited to English‐language websites, which could introduce cultural biases. Finally, the context in which the study was conducted may have contributed to defensive interpretations among investigators. To minimize potential bias, the fluoride messages were presented without context, and investigators were blinded to whether the messages were classified as correct or incorrect at the time of analysis. This blinding was possible because the primary study had been accepted for publication but was not yet publicly available during data analysis.

In conclusion, online fluoride misinformation is predominantly based on false attribution. However, it is important not to underestimate the deliberate use of scientific references to manipulate audiences, as demonstrated through quote mining techniques. These results contribute to understanding how misinformation is constructed to persuade audiences about supposed harmful effects of fluoride.

## 5 Clinical Relevance

### 5.1 Scientific Rationale for Study

Fluoride misinformation may shape public perception, drive anti‐fluoridation movements, and influence political decisions and treatment choices in dental practice.

### 5.2 Principal Findings

Online fluoride misinformation is primarily based on false attribution. Nonetheless, it is crucial not to underestimate the potential for malicious agents to manipulate fluoride‐related information using scientific references to serve their personal agendas, as demonstrated by instances of quote mining.

### 5.3 Practical Implications

The findings can help elucidate how authors construct messages to convince their audiences of supposed harmful effects of fluoride.

## Author Contributions

**Matheus Lotto:** conceptualization, methodology, formal analysis, investigation, writing – original draft, visualization. **Caio Sampaio, Olívia Santana Jorge**, and **Juliano Pelim Pessan:** investigation, formal analysis, writing – review and editing. **Thiago Cruvinel:** conceptualization, methodology, formal analysis, investigation, writing – review and editing, supervision, project administration.

## Funding

This study was supported by the São Paulo Research Foundation (Grant #2019/27242‐0 and #2023/11709‐2).

## Conflicts of Interest

The authors declare no conflicts of interest.

## Data Availability

This study analysed data from a primary investigation. The data that support the findings are openly available on Figshare at https://doi.org/10.6084/m9.figshare.c.5599266.v1.
